# Simulated gastrocnemius traction alters interfragmentary motion in Hoffa fracture fixation

**DOI:** 10.1007/s00068-025-03007-1

**Published:** 2025-11-14

**Authors:** Marianne Hollensteiner, Marlene Stallinger, Christof Hofmann, Mischa Mühling, Markus Greinwald, Sabrina Sandriesser, Dirk Baumeister, Peter Augat

**Affiliations:** 1 Institute for Biomechanics, BG Unfallklinik Murnau, Prof. Küntscher Str. 8, 82418 Murnau, Germany; 2 Institute for Biomechanics, Paracelsus Medical University Salzburg, Strubergasse 21, Salzburg, 5020 Austria; 3 Medical Engineering and Applied Social Sciences, Upper Austria University of Applied Sciences, Garnisonstr. 21, Linz, 4020 Austria; 4 Department of Trauma Surgery, BG Unfallklinik Murnau, Prof. Küntscher Str. 8, 82418 Murnau, Germany; 5 Institute for Biomechanics, BG Unfallklinik Murnau and Paracelsus Medical University Salzburg, Prof. Küntscher Str. 8, 82418 Murnau, Germany

**Keywords:** Hoffa fracture, Distal femur, Muscle pull simulation, Fracture fixation, Interfragmentary motion

## Abstract

**Background:**

Hoffa fractures remain biomechanically challenging due to their intra-articular location and limited fixation surface. The influence of posterior muscle forces—particularly from the gastrocnemius—on interfragmentary motion has not been adequately addressed in previous experimental studies. This study aimed to assess the impact of simulated gastrocnemius traction on interfragmentary motion in Hoffa fracture fixation.

**Methods:**

Patient-specific synthetic femora with anatomically realistic type I Hoffa fractures were manufactured from CT data using validated polyurethane-based materials. High-strength-fiber loops were embedded at the anatomical gastrocnemius insertion sites to simulate posterior muscle traction. Eight specimens with and eight without simulated gastrocnemius force (300 N constant pull) were tested under progressively increasing cyclic axial loading. Interfragmentary motion was captured via 3D motion tracking and analyzed for displacement and rotation.

**Results:**

Specimens with simulated muscle force exhibited significantly altered motion patterns compared to controls. Muscle traction reversed the direction of gap opening, increased gap twisting at higher loads (up to − 3.0°, *p* ≤ 0.005), and modified shear displacement and localized gap expansion. Despite these differences in fragment kinematics, no significant differences in construct failure load were observed (*p* = 0.599).

**Conclusion:**

Simulated gastrocnemius traction substantially influences interfragmentary motion in Hoffa fractures under axial load, even in the absence of changes in failure load. This study presents a novel test setup combining patient-specific fracture morphology and anatomically integrated muscle simulation, providing a transferable and physiologically relevant platform for future biomechanical investigations of distal femur fractures.

## Introduction

Hoffa fractures are coronal plane fractures of the femoral condyle, accounting for about 1% of all femoral fractures [1] often resulting from high-energy trauma. The lateral condyle is more frequently affected, representing approximately 85% of Hoffa fractures [2].

Open reduction and internal fixation is the preferred treatment for Hoffa fractures [2, 3]. Lateral Hoffa fractures are typically stabilized using cannulated screws, inserted either anterior-to-posterior or posterior-to-anterior, depending on the fracture configuration [2–4]. In cases of reduced bone quality or comminuted fragments, additional stabilization using an anti-glide plate can enhance construct stability.

Hoffa fractures are challenging to treat due to their intra-articular location and inherent biomechanical instability, characterized by limited surface area for surgical fixation, susceptibility to posteriorly directed shear forces, and the absence of stabilizing soft-tissue envelopes [5–7]. A critical yet often overlooked factor influencing the stability of Hoffa fractures is the force exerted by the gastrocnemius muscle, whose contraction can displace fracture fragments and compromise alignment and fixation stability [8]. The heads of the gastrocnemius originate from the medial and lateral femoral condyles and lie over the typical fracture site for Hoffa fractures. This anatomical relationship suggests that muscle contractions contribute to fragment displacement and may impede fracture healing [2, 4, 9].

Although muscle forces play a crucial biomechanical role in osteosynthetic fracture fixation, they are rarely integrated into in vitro experimental studies in general and in distal femur fractures, in particular. Biomechanical test setups often use synthetic or cadaveric bone models without consideration of muscle loads, limiting their ability to replicate the biomechanical behavior observed in vivo [10, 11]. Cadaver models are anatomically accurate but lack reproducibility due to technical complexity [12, 13]. Commercial bone surrogates often lack the mechanical integrity to support physiologic muscle load integration [14, 15].

Muscle-equivalent loading has been considered to some extent in biomechanical tests of proximal femur and femoral shaft fractures [15]. However, such approaches have not yet been adopted for distal femur or Hoffa fractures. The complexity of accurately simulating muscle forces in vitro and the challenges associated with replicating the dynamic interactions between muscles and bone likely contribute to this gap.

Across experimental and computational models, the role of muscle loading has been largely neglected [7]. Consequently, there is a need for further experimental research that integrates muscle forces to enhance the physiological relevance of biomechanical assessments in distal femur fractures.

Building on this rationale, the present study aimed to evaluate the biomechanical influence of simulated gastrocnemius muscle force on fixation stability in a standardized lateral Hoffa Type I fracture model treated with three anterior-to-posterior cannulated screws. We hypothesized that simulated gastrocnemius traction would significantly alter interfragmentary motion compared to controls without muscle force, even if ultimate failure loads remained unaffected.

## Materials and methods

### Specimen preparation

**Fig. 1:**
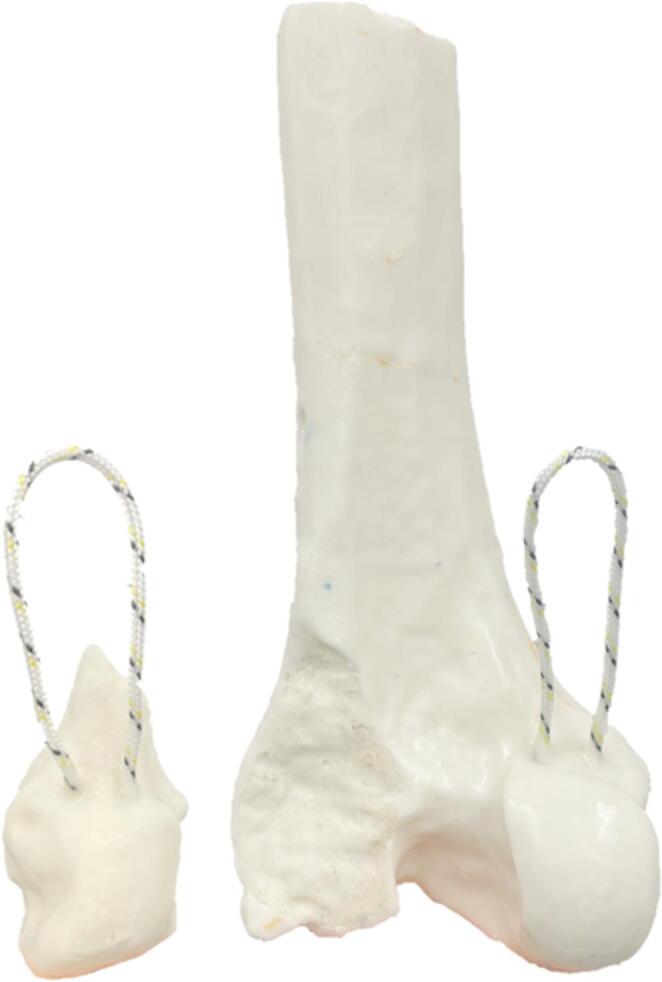
Distal PuReBone femur with a realistic lateral Hoffa fracture. UHMWPE loops simulate the insertion of the lateral and medial gastrocnemius muscles for applying physiologic muscle pulls

Previously developed and validated synthetic femur models, made from polyurethane resins and derived from computed tomography data, were used to generate a custom made a distal femur model that includes a lateral Hoffa fracture [14, 16]. Computed tomography (CT) scans of a human femur with a real, clinically observed lateral Hoffa fracture (Letenneur Type I, Fig. 1) were segmented using dedicated medical image processing software (D2P Professional v1.03, 3D Systems GmbH, Mörfelden-Walldorf, Germany). Both the cortical and trabecular bone were segmented separately to preserve the anatomical fidelity of the fracture morphology. The segmented models were 3D-printed (Raise3D N2 Plus printer, Raise 3D Technologies Inc., Irvine, CA, USA) to serve as master molds. Based on these master models, silicone molds were fabricated for the cortical and trabecular compartments. Using these molds, synthetic femora were cast from polyurethane-based resins, with the cortex consisting of a mineral-filler enriched polyurethane formulation, whereas the trabecular compartment—including that of the Hoffa fragment—was fabricated using mineral-filled edited cellular polyurethane foam. The material formulations have previously been validated against human bone in terms of morphology and mechanical properties (PuReBone [14, 16]),. Furthermore, a tibia was fabricated using the same manufacturing approach, based on the segmented CT data of the morphologically matching contralateral tibia. To provide a consistent and mechanically stable counter-bearing in the test setup, tibiae were produced using only the cortical material formulation, without a trabecular component. Furthermore, custom menisci were produced from hard silicone rubber (Sorta Clear 40, Smooth On Inc., Macungie, PA, USA) with a Shore Hardness A40. The menisci - cast in molds with an anatomical shape and a uniform thickness of 6 mm - approximated the peripheral proportion of human menisci [17]. The silicone menisci ensured physiologically relevant load transmission across the joint space. The silicone menisci were not intended to replicate native tissue mechanics but to compensate for irregular bony surfaces from patient-derived CT geometry, thereby preventing unwanted sliding and ensuring consistent load transmission. Prior to testing, the setup was verified to confirm reliable force transfer to the tibia.

To simulate physiological loading conditions, loops based on ultra-high-molecular-weight polyethylene (UHMWPE) fibre (Dyneema SK99, Sagola High Performance Line, Seacsub S.p.a, San Colombano Certenoli, Italy) were embedded into the posterior femoral condyles during casting, corresponding to the anatomical insertion sites of the lateral and medial gastrocnemius muscles (Fig. 1). Specifically, one UHMWPE loop was embedded in the intact medial femoral condyle, and a second loop was integrated directly into the Hoffa fracture fragment of the lateral condyle. The material was chosen due to its high tensile strength, low elongation, and high fatigue resistance [18], making it well-suited for applying physiological muscle forces. These embedded loops allowed for external connection to a loading system simulating muscular traction forces acting on the distal femur, including direct load application to the Hoffa fragment.

### Fracture treatment

**Fig. 2:**
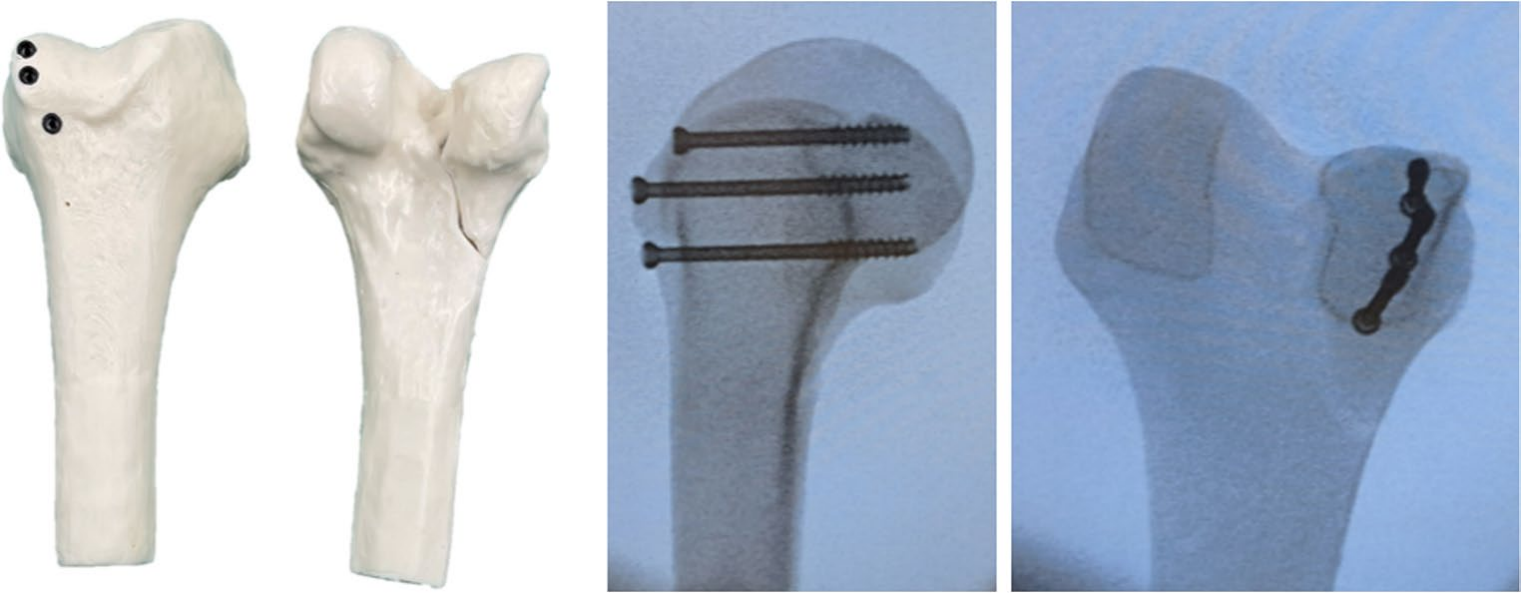
Distal femur with a lateral Hoffa fracture treated using three parallel 5 mm cannulated screws inserted in an anterior-to-posterior direction (left). Postoperative radiograph in medial to lateral (middle) and anterior to posterior (right) projection

All Hoffa fractures were fixated by a single experienced orthopedic trauma surgeon in order to ensure consistency and procedural reproducibility. For fixation, three 5 mm cannulated screws (ASNIS III, Stryker, Kalamazoo, MI, USA) were inserted from anterior to posterior, following the manufacturer’s surgical technique guide. All screws were advanced to the posterior subchondral region of the condyle. Although the synthetic model does not contain cartilage, intraoperative fluoroscopy confirmed screw placement corresponding to the posterior load-bearing zone of the human condyle (Fig. 2).

### Mechanical testing

**Fig. 3:**
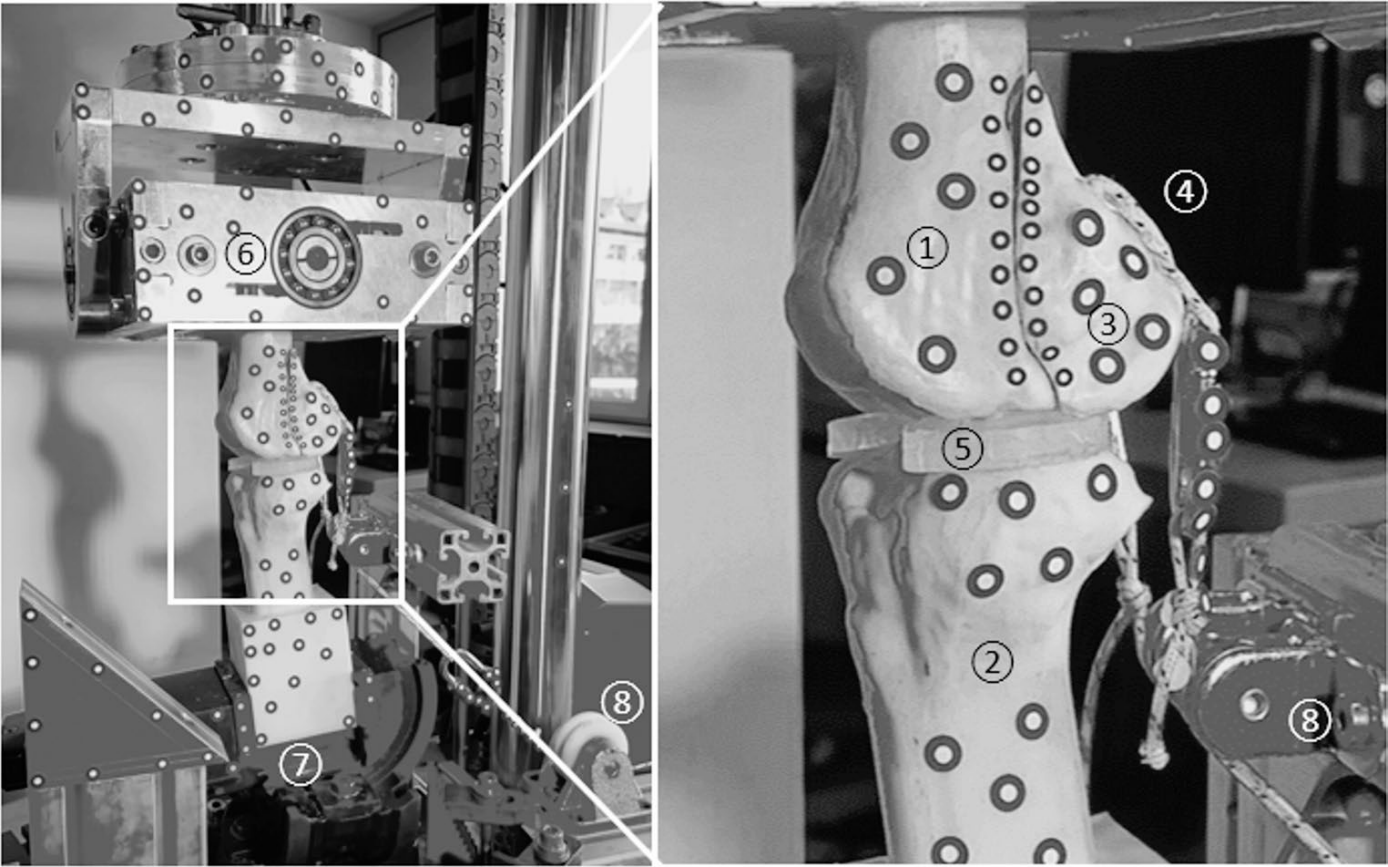
Test setup for axial loading with simulated muscle force. The overview (left) shows the mounted specimen in the testing machine; the inset (right) highlights the region of interest. (1) distal femur, (2) proximal tibia, (3) Hoffa fragment, (4) embedded UHMWPE loops for muscle force application, (5) meniscus made of silicone, (6) cardan joint, (7) rigid fixation to the machine baseplate, (8) deflection pulleys for applying the tensile muscle load

For biomechanical evaluation, each synthetic femur was mounted in a testing machine (E3000, Instron Structural Testing GmbH, Darmstadt, Germany) in physiologic orientation at terminal stance during gait [19]. Although the actuator applied a vertical force, the oblique femoral shaft alignment relative to the load axis resulted in both axial compression across the tibiofemoral articulation and concomitant frontal-plane bending. This setup therefore reproduces the predominant joint loading condition during terminal stance while allowing for standardized cyclic testing. The femur was proximally embedded with fast curing resin (GP010 casting resin, Gößl & Pfaff GmbH, Karlskron, Germany) in a cardan coupling used to allow self-alignment and to minimize shear forces during loading. The distal femur was articulated against the tibia, which was embedded and rigidly fixed to the machine base. The custom-made silicone menisci were interposed to ensure physiological load distribution and surface conformity (Fig. 3).

Axial cyclic loading was applied dynamically at 2 Hz, starting from an initial joint contact force of 500 N. For specimens with simulated muscle force, an external posterior pull of 300 N was applied via embedded UHMWPE loops at the gastrocnemius origins, connected through carabiners and redirection pulleys to a constant weight. The direction of pull was anatomically aligned to replicate the physiological force vector of the gastrocnemius muscle, acting from the posterior femoral condyles toward the calcaneus. To maintain equivalent resultant joint force across groups, these specimens were subjected to an initial machine load of 200 N, while control specimens without muscle force received 500 N directly from the testing machine. This ensured that the net joint contact force was standardized at 500 N for all specimens at the beginning of loading. The load was progressively increased by 0.05 N per cycle until either structural failure or a displacement threshold of 10 mm was reached.

### Assessment of interfragmentary motion

To capture interfragmentary motion, adhesive markers were attached to the femur, Hoffa fragment, and setup (Fig. 3). A 3D optical motion capture system (ARAMIS Adjustable, Zeiss GOM metrology GmbH, Braunschweig, Germany) was used to track spatial displacements and rotations. Those were calculated based on a global coordinate system defined with y-axis oriented along femurs shaft axis, x-axis aligned with anterior-posterior axis and z-axis aligned with medio-lateral axis. Evaluated parameters included gap opening, defined as rotation of the Hoffa fragment around the medio-lateral axis [20], gap twisting, defined as rotation of the Hoffa fragment around the antero-posterior axis [20], shear displacement at the fracture site in frontal plane, and axial displacement of the Hoffa fragment along the femoral shaft axis. Furthermore, fracture gap expansion was assessed at two defined positions (proximal, distal) in anterior-posterior axis.

### Data analysis

All data were analyzed using SPSS (SPSS Statistics v.26, IBM, Amonk, USA). Normal distribution of continuous variables was assessed using the Shapiro–Wilk test. For each biomechanical parameter group comparisons were performed between specimens with and without simulated muscle force (*n* = 8 per group). As several parameters were not normally distributed, group comparisons were consistently performed using the non-parametric Mann–Whitney *U* test. Statistical significance was defined as *p* < 0.05. Results are reported as mean ± standard deviation (SD) for descriptive purposes.

Interfragmentary motion was initially assessed in 50 N increments from 500 N to 750 N and subsequently in 250 N steps up to 1750 N. Load levels beyond 1750 N were excluded from analysis due to specimen failure. To enhance clarity and improve axis scaling in figures, only selected load levels (500 N, 1000 N, 1250 N, 1500 N, and 1750 N) are shown in the graphs, as intermediate values did not yield relevant group differences. Statistical comparisons, however, were performed across all load levels. Load to failure was assessed at the point of actual mechanical failure during testing, which varied between specimens. Mechanical failure was defined as either (i) fracture of the synthetic bone, (ii) failure or pull-out of the screw–bone construct, or (iii) an axial displacement exceeding 20 mm.

## Results

### Load to failure

There was no statistically significant difference in ultimate failure load between specimens with simulated muscle force (2314 ± 273 N) and those without muscle force (2298 ± 150 N; *p* = 0.599) suggesting no relevant impact of simulated muscle force on ultimate failure load.

### Interfragmentary motion

**Fig. 4:**
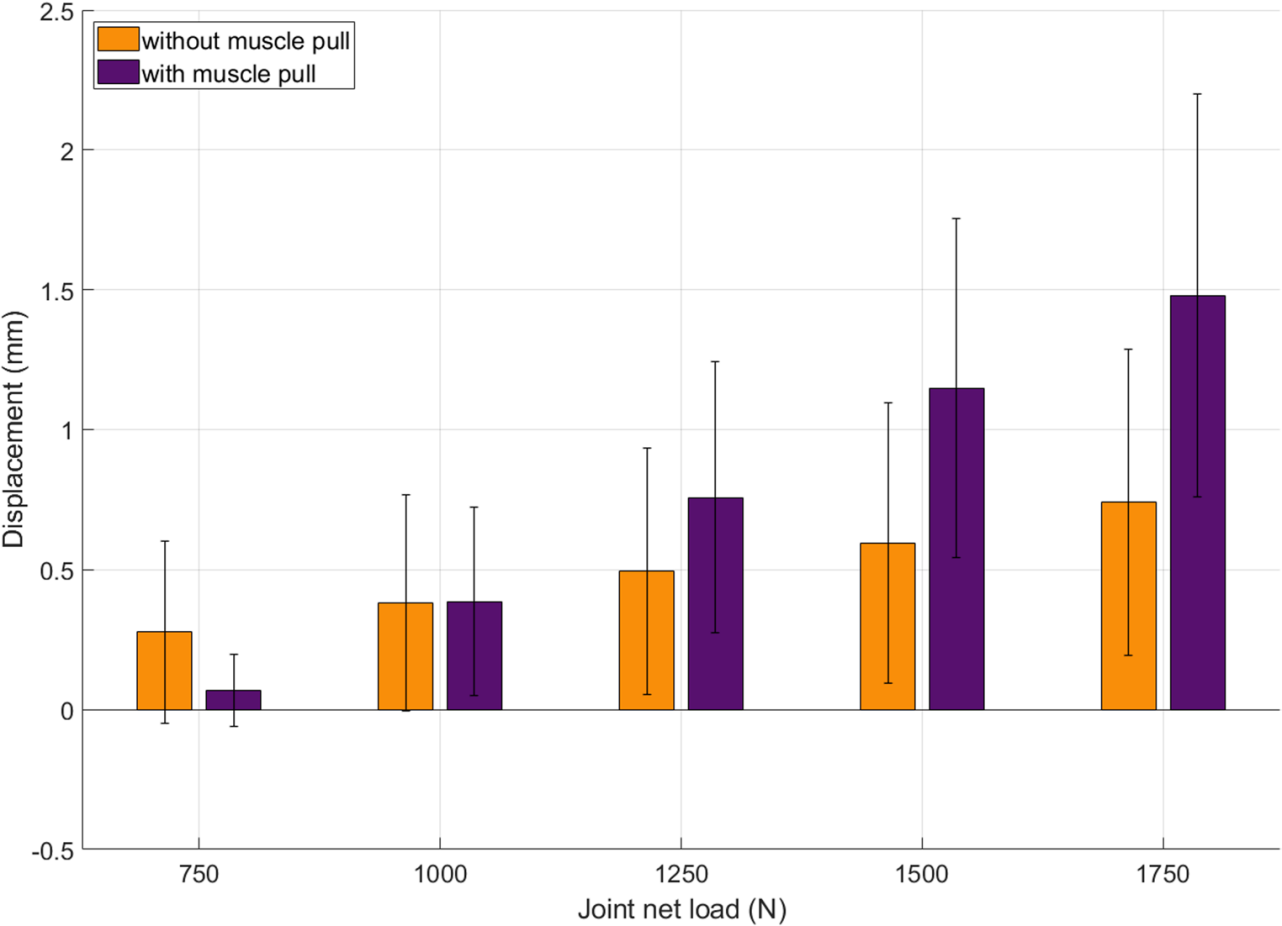
Axial displacement of the Hoffa fragment under increasing cyclic axial loading in specimens with (purple) and without (orange) simulated gastrocnemius muscle force. Positive displacement values indicate cranial migration of the fragment relative to the femoral shaft. Error bars represent standard deviation

Axial displacement of the Hoffa fragment remained below 1.5 mm in both groups throughout the loading protocol, indicating high primary stability of the screw fixation (Fig. 4). In specimens with simulated gastrocnemius traction, axial displacement was caudal (negative) below and crossed zero at approximately 700 N. At 1000 N, values were comparable between groups with and without muscle traction, whereas at higher loads cranial migration became significantly greater with muscle traction (1500 N: *p* = 0.038; 1750 N: *p* = 0.028).

**Fig. 5:**
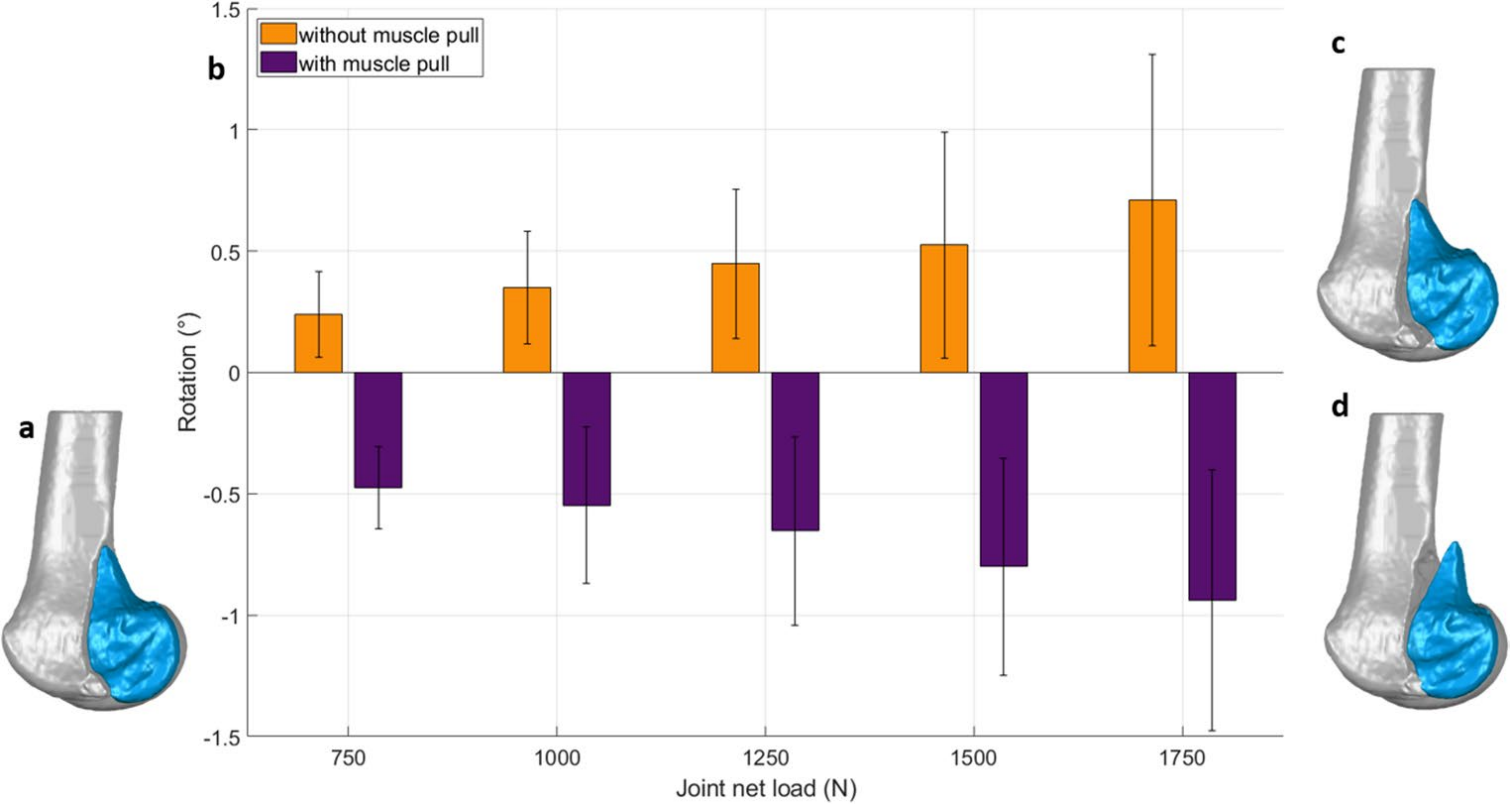
Gap opening of the Hoffa fragment under increasing cyclic axial loading, quantified as rotation around the medio-lateral axis. (**a**) 3D model of the lateral Hoffa fracture with the fragment in situ. (**b**) Bar graph of measured fragment rotation: specimens without simulated muscle force (orange) showed consistent positive rotation (distal opening), while specimens with gastrocnemius traction (purple) rotated negatively across all load levels (proximal opening). Error bars represent standard deviation. (**c**) 3D schematic illustrating distal opening of the fracture gap (without muscle force) (**d**) 3D schematic illustrating proximal opening of the fracture gap (with simulated gastrocnemius traction). (c) and (d) shown in an exaggerated manner for visualization

Rotational displacement of the Hoffa fragment around the medio-lateral axis (gap opening, Fig. 5) remained below 1.5° in both groups throughout the loading protocol. From 750 N upward, specimens with simulated gastrocnemius traction consistently showed significantly different rotation patterns compared to controls (*p* < 0.001). While absolute values were low, the direction and magnitude of rotation were distinctly influenced by the muscle force, which reversed the typical tilt induced by axial compression.

**Fig. 6:**
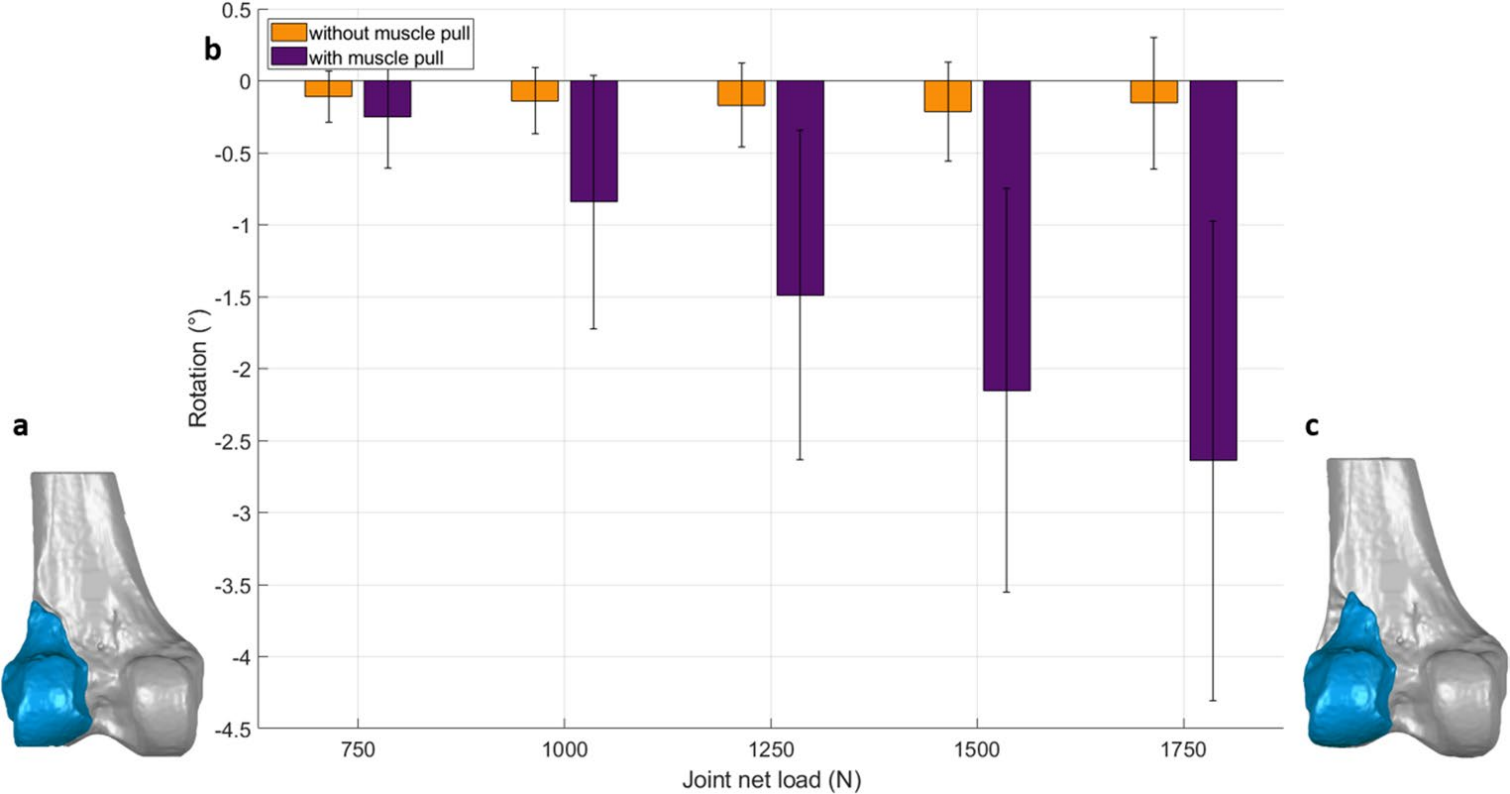
Gap twisting of the Hoffa fragment under increasing cyclic axial loading, measured as rotation around the antero-posterior axis. (**a**) 3D model of the lateral Hoffa fracture with the fragment in situ after fixation. (**b**) Bar graph of measured fragment rotation: both groups showed consistent rotational direction, but specimens with simulated gastrocnemius force (purple) exhibited markedly greater twisting at higher loads compared to controls without muscle force (orange). Error bars represent standard deviation. (**c**) 3D schematic illustrating the twisting motion of the Hoffa fragment (exaggerated for visualization)

Rotation of the Hoffa fragment around the antero-posterior axis (gap twisting) was negative in both groups across all load levels, indicating a consistent rotational direction (Fig. 6). However, from 1000 N upward, specimens with simulated gastrocnemius traction showed a significantly greater magnitude of twisting compared to controls, with angles reaching − 2.5° to − 3.0° at 1750 N (*p* ≤ 0.005).

**Fig. 7:**
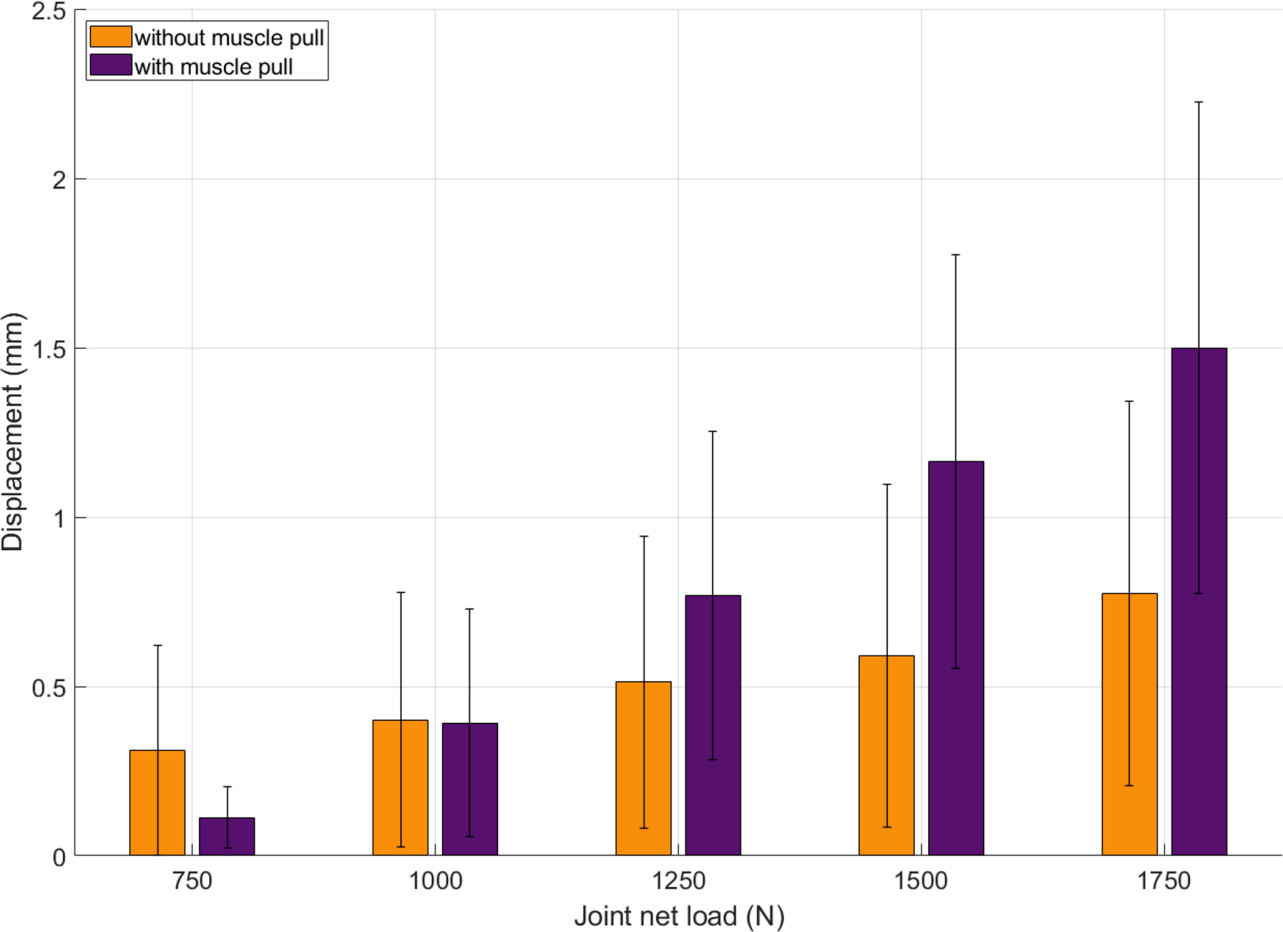
Shear displacement of the Hoffa fragment in the frontal plane. Up to 750 N, muscle-loaded specimens (purple) showed reduced displacement compared to controls (orange). Above 1000 N, shear increased markedly in the muscle group, possibly due to constraint effects from constant posterior traction. Error bars represent standard deviation. The dashed line marks the change in load step size

Shear displacement of the Hoffa fragment in the frontal plane remained below 1.5 mm across all load levels, but group differences became apparent at higher loads (Fig. 7). While values were comparable up to 1000 N (*p* = 0.721), specimens with simulated gastrocnemius traction showed a marked increase beyond this point, reaching up to 1.5 mm at 1750 N. Significant differences were observed at 1500 N (*p* = 0.050) and 1750 N (*p* = 0.005).

**Fig. 8:**
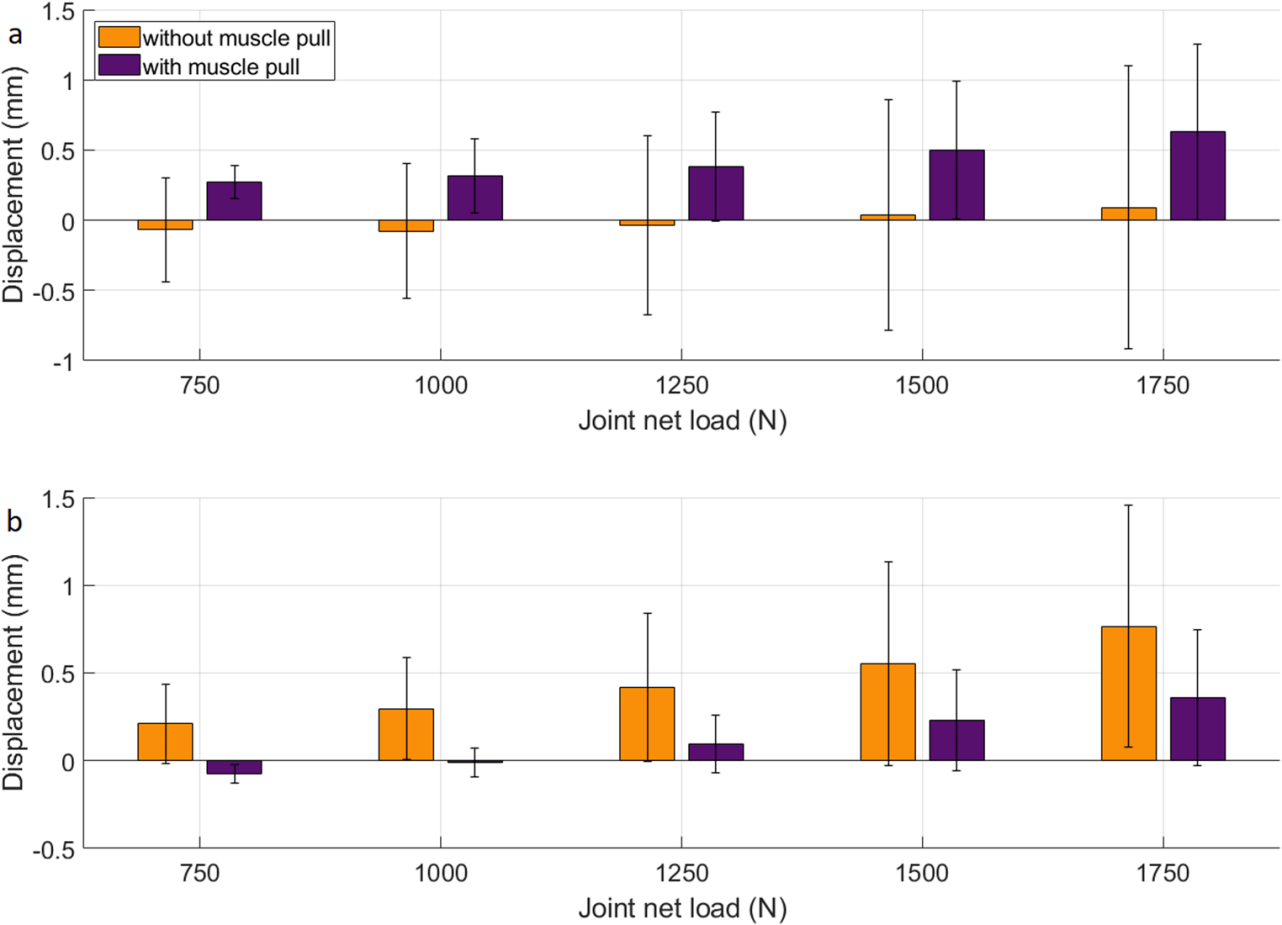
Gap expansion at two locations along the fracture line under increasing cyclic axial loading. (**a**) Proximal point. (**b**) Distal point. Error bars represent standard deviation. Positive values indicate motion in posterior direction; negative values motion in anterior direction

Gap expansion along the fracture line differed significantly between groups from 750 N upward at both the proximal and distal fracture edges (Fig. 8). In specimens with simulated gastrocnemius traction, displacement directions were consistently reversed compared to controls, reflecting a load-dependent redistribution of motion. Statistical analysis confirmed highly significant group differences at both locations (*p* < 0.001).

## Discussion

This study demonstrated that simulated gastrocnemius muscle traction significantly influences the interfragmentary motion of anatomically reduced Hoffa Type I fractures under physiological knee joint loading. In specimens with simulated Gastrocnemius muscle pull a consistent and statistically significant differences in fragment kinematics was observed compared to controls without muscle pull. While ultimate failure loads remained similar between both groups, muscle traction altered the direction and magnitude of gap opening, increased gap twisting at higher loads and affected shear displacement and gap expansion along the fracture line.

These distinct kinematic alterations can be biomechanically explained by the anatomical location and direction of the applied muscle force. Analyzing each motion parameter in detail allows further insight into how gastrocnemius traction modifies fragment behavior under increasing joint compression.

In axial displacement, specimens without muscle traction showed gradual cranial migration of the Hoffa fragment with increasing load, caused by joint compression. With simulated gastrocnemius traction, the fragment initially displaced caudally at low loads (550–700 N), reflecting the dominant posterior pull. At 1000 N, axial compression balanced this effect, and from 1500 N upward cranial migration exceeded that of controls without muscle traction. This can be explained by a shift of the tibiofemoral contact anteriorly under compression, coupled with posterior muscle-induced rotation, which transformed into an additional cranial component of motion.

In gap opening, the direction of fragment rotation was reversed: machine loading induced a distal opening of the fracture gap, while muscle traction caused a posterior tilt, leading to proximal gap opening. This highlights the antagonistic effect of posterior muscle force acting above the screw fixation and illustrates its role as a rotational driver around the medio-lateral axis.

Gap twisting revealed further asymmetrical loading effects: while both groups rotated in the same direction, the muscle-loaded specimens showed significantly greater rotation at higher loads. This suggests that the convergence of the two simulated gastrocnemius heads into a single posterior traction line generated a torsional moment around the antero-posterior axis—an effect absent in the control group. Although the observed rotational differences remained small (< 3°), studies have shown that even minor condylar deviations can alter joint mechanics [21]. This underlines that posterior muscle forces may introduce clinically relevant micromotions at the articular surface, even when the fixation construct appears mechanically stable. From a rehabilitation perspective, this supports careful progression of weight-bearing and physiotherapy protocols in the early postoperative phase, as posterior muscle activation could subtly affect joint loading and fragment kinematics. Even small steps or gaps in reduction can lead to altered joint mechanics, post-traumatic arthritis, and loss of function, leading authors to recommend early surgical treatment and meticulous reduction for all displaced Hoffa fractures, regardless of the specific degree of malrotation or angular discrepancy [4]. Shear displacement also reflected a load-dependent behavior: at low loads, muscle traction reduced micromotion, acting like a tension band. However, at higher loads, the same constant force interfered with joint compression and created a lateral shift of the fragment, increasing shear displacement beyond that of the control group.

Finally, the analysis of fracture gap expansion confirmed the location-specific action of muscle traction. Without muscle force, the fragment tilted posteriorly under axial loading, as evidenced by gap opening proximally and dorsal closure distally. In the muscle-loaded group, this pattern was inverted: proximal pull from the gastrocnemius caused dorsal opening, while distal motion was reduced. These findings demonstrate that posterior muscle forces act neither purely stabilizing nor destabilizing, but rather introduce spatially differentiated mechanical effects that depend on insertion point, force direction, and load magnitude. Such interactions highlight the biomechanical relevance of simulating muscle forces in fracture models, especially when evaluating interfragmentary motion under near-physiological conditions. These findings not only underline the biomechanical relevance of posterior muscle traction but also emphasize the methodological novelty of the present model.

The observed muscle-induced alterations in fragment motion raise important considerations for the design and optimization of fixation constructs in Hoffa fractures. While the use of three anterior-to-posterior screws provided stable fixation under loading, the directional effects of muscle traction - particularly the rotational and shear components - suggest that certain fragment regions are more vulnerable to displacement depending on muscular engagement. The screw configuration employed in this study, with three anterior-to-posterior screws in a nearly colinear row, reflects both clinical practice and a frequently reported construct in the literature [2, 20, 22]. This placement provides compression across the coronar fracture line, safe anterior access without risk to posterior neurovascular structures, and primary rotational stability. Nevertheless, our findings demonstrate that posterior gastrocnemius traction can still induce measurable fragment rotations, indicating that muscle loading may challenge this conventional construct. To address this, a follow-up study evaluating screw–plate constructs with broader muscle force application and higher simulated gastrocnemius traction to determine whether these strategies can provide improved control of rotation and shear is conducted. These findings may also inform implant design by highlighting zones of increased mechanical demand that arise not from joint loading alone, but from the interaction between bone geometry, muscle force vectors, and fixation placement.

To our knowledge, this is the first experimental study to simulate posterior muscle traction in a lateral Hoffa fracture model using a patient-specific synthetic femur, anatomically segmented fracture morphology, and embedded muscle insertion points. While previous biomechanical studies have neglected muscular influences, the present setup allows for controlled, anatomically plausible simulation of gastrocnemius force, directly applied to the fracture fragment via cast-in loops. This test concept overcomes the limitations of osteotomized epoxy models and expands the methodological toolbox for biomechanical research in intra-articular distal femur fractures. This approach offers a new and transferable platform for future investigations of muscle–implant–bone interactions under near-physiological loading conditions.

The comparability of results between studies is limited due to differences in experimental setup, including fracture generation, fixation configuration, loading protocol, and boundary conditions. Several previous studies have investigated Hoffa fracture fixation under axial loading, but did not account for muscular forces. Pires et al. conducted monotonic axial loading on osteotomized epoxy-based Sawbones using different fixation methods. Their specimens with AP screw fixation failed at approximately 600 N, though displacements were not reported in such detail [22]. Sun et al. tested a similar Sawbones model with two AP screws and localized axial loading, reporting a notably higher failure load of approximately 1800 N [23]. Peez et al. reported axial displacements of approximately 1.8 to 2.0 mm, gap opening of approximately 1.7°, and twisting up to 2° at a failure load of 434 N in cadaveric specimens with two anterior-to-posterior screws and punch-based axial loading applied directly to the fractured condyle [20]. In the present study, such magnitudes of motion were not observed even under substantially higher cyclic loading conditions (up to 1750 N). In both test groups—with and without simulated gastrocnemius traction—axial displacement remained below 0.5 mm, gap opening below 1.5°, and twisting below 2.5°. However, while direct mechanical comparison is not feasible, the similar range of motion values provides a reference frame. More importantly, within the current study, the addition of simulated muscle force consistently influenced interfragmentary motion: it reversed the direction of gap opening, reduced shear displacement at low loads, and introduced characteristic torsional effects. These results underscore the biomechanical relevance of incorporating muscle-equivalent loading in fracture models—a parameter not considered in previous studies.

From a clinical perspective, the present findings emphasize the potential role of posterior muscle forces—particularly from the gastrocnemius—in modulating interfragmentary motion during early weight bearing. While all specimens were fixated with three large-diameter screws in an anatomically aligned configuration, the group with simulated muscle force exhibited direction-specific changes in fragment kinematics, even without differences in failure load. These results suggest that muscular traction may either stabilize or destabilize fracture fragments depending on load level. This could be particularly relevant during rehabilitation, where involuntary muscle activation or functional loading may introduce rotational or shear stresses at the fracture site. Surgeons should be aware that muscle forces—even if not overtly visible—can influence fracture behavior postoperatively. This may be especially relevant in intra-articular fractures like the Hoffa lesion, where fragment geometry and soft-tissue balance are delicate.

Despite the strengths of the presented approach, several methodological limitations must be acknowledged.

First, the muscle force was applied as a constant posterior traction of 300 N, which does not reflect the dynamic and time-varying activation patterns of the gastrocnemius during physiological movement. This value was selected as a standardized, conservative baseline to ensure reproducibility across specimens while remaining within the range of literature-reported forces during walking. The femur and tibia were positioned in terminal stance alignment, a physiologically relevant and mechanically demanding phase of gait where gastrocnemius activity peaks. Although patients are initially advised to avoid full rollover motion after distal femur fractures, clinical experience shows that many compensate by “hopping” with crutches [24]. Nevertheless, restoration of terminal stance is a central rehabilitation goal and essential for regaining normal gait. Modeling this phase therefore represents a clinically relevant target scenario that fixation constructs must ultimately withstand.

In vivo muscle forces, however, are difficult to quantify directly [25] and show substantial variability between individuals [26]. Current estimates are derived from inverse dynamics and musculoskeletal simulations, such as OpenSim or AnyBody, which differ in their representation of muscle geometry, recruitment strategies, and anthropometric scaling [27]. Consequently, literature values for gastrocnemius force during gait vary considerably, ranging from several hundred to over 1500 N depending on the model and gait cycle phase. For example, inverse dynamics approaches typically estimate peak gastrocnemius forces of 300 to 600 N during walking, whereas musculoskeletal simulations predict higher values of 800 to 1200 N, and experimental techniques report maximum force-generating capacities exceeding 1500 N [28]. The 300 N applied here was therefore selected as a standardized, conservative baseline rather than a physiological maximum, providing reproducibility across specimens while acknowledging modeling uncertainty.

Second, while the simulated muscle forces were applied at anatomically accurate insertion points using embedded UHMWPE loops, the force transmission was limited to two discrete attachment sites. This simplification does not fully replicate the broad, distributed nature of physiological muscle insertions and may affect the spatial characteristics of load application.

Consequently, literature values for gastrocnemius force during gait vary considerably, ranging from several hundred to over 1500 N depending on the model and gait cycle phase. These discrepancies can be attributed to both methodological differences and gait-phase-dependent force variations. For example, inverse dynamics approaches typically estimate peak gastrocnemius forces of 300 to 600 N during walking, whereas musculoskeletal simulations using OpenSim or AnyBody predict higher values of 800 to 1,200 N due to differing assumptions regarding muscle recruitment strategies and tendon compliance [28]. Experimental techniques yield additional variability: ultrasound-based measurements during walking suggest lower forces around 400 N at moderate speeds, while electrical stimulation experiments indicate maximum force-generating capacities exceeding 1,500 N [28]. Moreover, force magnitudes vary substantially throughout the gait cycle, with mid-stance values averaging 300 to 500 N and peak forces of 800 to 1,700 N typically occurring during terminal stance or push-off, depending on the modeling approach [28–30]. The 300 N applied here was therefore selected as a standardized, conservative baseline rather than a physiological maximum, providing reproducibility across specimens while acknowledging modeling uncertainty.

To ensure consistent joint contact and compensate for surface irregularities between femur and tibia, a silicone material was used to approximate meniscal geometry. While this provided geometric conformity and stable load distribution, it does not replicate the mechanical properties of the native meniscus. The material used for simulating the meniscis, Sorta Clear 40, has a tensile strength of approximately 5.5 MPa and a Shore A hardness of 40 [31], whereas human menisci exhibit circumferential tensile strength up to 300 MPa, largely due to their organized collagen fiber structure [32]. Similarly, physiological meniscal compression stresses range between 1 and 3 MPa [33], which is only partially comparable to the compressive behavior of the silicone used. Thus, the material served only to provide form-fit contact, not to replicate true meniscal function or load distribution.

Finally, polyurethane-based synthetic femora were used in this study. Although these models were anatomically segmented from patient CT data and mechanical properties were previously validated to mimic the mechanical and morphological properties of human bone [14, 16], they do not fully replicate the heterogeneous structure, viscoelasticity, or biological response of living bone. Cadaveric specimens could offer more realistic tissue behavior, but integrating reproducible and anatomically anchored muscle force application in human bone remains challenging. Similarly, commercially available epoxy-based synthetic bones offer high reproducibility but are poorly suited for simulating muscle forces; muscle integration typically requires screws or eyelets, introducing non-physiological levers or stress concentrations. In contrast, the casting-based method used here allowed for anatomically accurate, integrated muscle insertion sites, offering a more physiologically plausible approach to simulate soft tissue interaction under load.

This study demonstrates that simulated gastrocnemius traction significantly alters the direction and magnitude of interfragmentary motion in type I Hoffa fractures under axial loading with differences in gap opening, gap twisting, shear displacement, and gap expansion, particularly at higher load levels. This highlights the ability of posterior muscle forces to modulate fragment kinematics independently of fixation strength. The presented test setup—with patient-specific fracture morphology and anatomically integrated muscle loading—introduces a novel and transferable platform for biomechanical evaluation. By enabling physiologically meaningful simulation of soft tissue–bone–implant interactions, this approach advances current experimental models and may improve the relevance of future in vitro studies for optimizing fracture fixation strategies.

## Data Availability

Data can be obtained on request from the corresponding author.

## Declarations

### Use of LLM, AI and machine learning tools

Artificial intelligence tools (DeepL.com and QuillBot.com) were employed to enhance the language quality of the manuscript.
